# The Missing Temporal Coordinate: A Framework for Biopsy Timestamping and Chrono-Biobanking in Precision Oncology and Longevity Medicine

**DOI:** 10.7759/cureus.119434

**Published:** 2026-11-10

**Authors:** Ivan Bivolarski, Martin Dimitrov, Tanio Stefanov, Yavor S Pavlov, Anita R Hvarchilkova-Baycheva, Stanislav Marangozov

**Affiliations:** 1 Medical Oncology, Integrated Oncology Centre, Burgas, BGR; 2 Department of General, Vascular, and Visceral Surgery, Greiz District Hospital, Greiz, DEU; 3 Department of Surgical Oncology, Burgasmed LTD, Burgas, BGR; 4 Pathology, University Multiprofile Hospital for Active Treatment “Burgasmed”, Burgas, BGR; 5 Medical Oncology, Comprehensive Cancer Center - Veliko Turnovo, Veliko Turnovo, BGR; 6 General and Thorax Surgery, University Multiprofile Hospital for Active Treatment “Burgasmed”, Burgas, BGR

**Keywords:** biopsy timestamping, biospecimen science, chrono-biobanking, circadian rhythms, geroscience, longevity medicine

## Abstract

Precision medicine increasingly relies on biospecimen analysis for diagnosis, prognostic assessment, treatment selection, and longitudinal monitoring. Established biobanking frameworks address collection methods, processing delays, fixation, and storage. Collection times may also be recorded, but documentation may be inconsistent, and timestamps may not be retained in research datasets or considered in subsequent analyses. Because several physiological processes vary across the day, sampling time may provide relevant context for interpreting selected molecular and cellular measurements.

In oncology, circadian regulation influences immune cell distribution, endocrine signaling, metabolism, and gene expression. Sampling time may therefore contribute to variability in selected blood-based biomarkers and molecular profiles. However, the magnitude and clinical relevance of these effects differ across analytes, tissues, and clinical settings and remain insufficiently characterized for many routinely used biomarkers.

Similar considerations apply to geroscience and longevity medicine, where some measurements arise from systems with daily variation. Whether this variability meaningfully affects specific epigenetic clocks, biological age estimators, or assessments of intervention response requires direct validation.

This narrative review examines evidence relevant to the temporal context of biospecimen collection and proposes a practical framework for timestamping and chrono-biobanking. Proposed core metadata include the local collection date and time, the applicable time zone or UTC (Coordinated Universal Time) offset when available, specimen type, anatomical site, and clearly distinguished processing timestamps. Additional information on fasting, medications, treatment exposure, sleep, and chronotype may be recorded according to the clinical or research question. Comparable collection windows may be useful for serial sampling when feasible and scientifically justified.

The proposed framework prioritizes systematic documentation within existing workflows. Integration with electronic health records, laboratory systems, and biobank databases could facilitate the capture and preservation of collection times without delaying necessary care. These data may support temporal stratification, sensitivity analyses, and statistical adjustment where study design and data permit. The framework’s feasibility and its effect on biomarker interpretation require empirical evaluation. Preserving sampling-time information provides a foundation for investigating its relevance to biomarker reproducibility and interpretation in precision oncology and longevity research.

## Introduction and background

Precision medicine increasingly relies on biospecimen analysis for diagnosis, risk stratification, treatment selection, and longitudinal monitoring. In oncology, tissue and liquid biopsies provide information about disease biology and predictive or prognostic biomarkers. Geroscience and longevity medicine similarly use molecular measurements to investigate biological aging and responses to interventions. The interpretation of these measurements depends on both analytical performance and the conditions under which specimens are collected.

Established biobanking frameworks address collection methods, processing delays, fixation, storage, and other preanalytical variables [1,2]. Although collection timestamps may be documented, recording practices may be inconsistent, and this information may not accompany specimens into research datasets or biomarker analyses. Reliable documentation is important because sampling time preserves biological context that may be difficult to reconstruct retrospectively.

Three distinct temporal variables should be distinguished: the clock time of specimen collection, the timing of sampling relative to treatment administration, and the interval between collection and specimen stabilization. These variables address different questions. Clock time provides a reference for investigating daily biological variation; treatment-relative timing indicates whether sampling occurred before or after a therapeutic exposure and, when applicable, the interval since treatment administration; and processing intervals describe preanalytical handling [1,2]. Recording only the laboratory receipt time cannot establish all three. For example, paired pretreatment and on-treatment samples collected at different hours may differ in both treatment exposure and sampling time, complicating interpretation of observed changes.

The circadian system generates approximately 24-hour rhythms through interconnected molecular feedback loops [3]. These rhythms influence gene expression across tissues [4,5] and contribute to metabolic, endocrine, and immune regulation. Mistimed sleep can disrupt the temporal organization of gene expression in human blood [6]. These findings are consistent with the broader role of circadian regulation in metabolic homeostasis and disease-related physiology [7]. A specimen may therefore reflect both disease-related features and temporal variation in cellular composition or molecular activity. Clock time provides a practical reference, although it does not directly measure internal circadian phase.

For time-sensitive biomarkers, differences in sampling time may complicate comparisons between patients or successive measurements. Circulating leukocyte populations show sleep- and circadian-dependent variation, partly reflecting rhythmic immune cell trafficking [8-10]. Cancer-associated measurements may also be affected: a study in breast cancer reported greater circulating tumor cell release during the resting phase in the investigated setting [11]. Such findings support preserving sampling-time information while recognizing that temporal sensitivity may vary between biomarkers, diseases, and clinical settings.

Similar considerations apply to aging research. DNA methylation-based clocks estimate biological aging [12,13], while other approaches incorporate transcriptomic, proteomic, metabolic, and inflammatory measurements. Daily variation in some contributing signals may influence interpretation, although its relevance requires validation for each estimator. Age-related changes in circadian organization provide a further rationale for retaining temporal context [14].

This narrative review examines evidence relevant to sampling time in tissue gene expression, circulating immune cells, liquid biopsy, and molecular measurements used in aging research. It distinguishes findings from human studies and experimental models from proposed applications that require clinical validation. Particular attention is given to the distinction between time-of-day associations and endogenous circadian regulation, the separation of biological variation from specimen handling effects, and the potential consequences for longitudinal biomarker comparisons. On this basis, the review proposes a practical framework for biopsy timestamping and chrono-biobanking, including minimum temporal metadata, additional contextual variables, and approaches to evaluating sampling-time effects. Its purpose is to outline how temporal annotation can support biomarker-specific validation and inform decisions about standardized collection schedules without delaying necessary clinical care.

Approach to literature selection and synthesis

This article is a narrative review. Relevant literature was identified through PubMed/MEDLINE, Scopus, Web of Science, and Google Scholar. The search focused on circadian and time-of-day variation in tissue gene expression, circulating immune cells, liquid biopsy, and molecular biomarkers of aging, as well as preanalytical variables and biobanking practices. Search terms included combinations of “circadian,” “time of day,” “sampling time,” “chrono-biobanking,” “liquid biopsy,” “circulating tumor cells,” “DNA methylation clock,” “preanalytical variables,” and “biomarker.”

Publications published between January 1, 1997, and September 1, 2026, were considered. Only English-language publications were considered. Publications were selected based on their relevance to the review’s objectives and included original human studies, experimental models, methodological papers, and relevant reviews.

Findings were compared descriptively across themes, considering study design, population or experimental model, biomarker, and, where reported, the timing of specimen collection. Human studies and experimental models were interpreted in their respective contexts. Differences in study design, biological context, and reported sampling schedules were considered when interpreting the consistency and applicability of findings. The evidence was synthesized narratively; no meta-analysis or quantitative pooling of effect estimates was performed.

## Review

The missing temporal coordinate in precision oncology

Precision oncology uses biospecimens to characterize disease biology and guide clinical decisions. Tumor tissue supports histological diagnosis, genomic profiling, protein expression assessment, and evaluation of the immune microenvironment. Blood and liquid biopsies provide complementary information through hematological indices, circulating molecules, immune cells, and tumor-derived components. Interpretation of these measurements may benefit from consideration of sampling time, particularly when the measured signals exhibit daily variation.

Tissue-based diagnostics appropriately emphasize cold ischemia, fixation, storage, and analytical methods [1,2]. Collection times may also be recorded, but documentation may be inconsistent, and receipt or processing timestamps may not always be clearly distinguished from the time of tissue removal. These timestamps describe different events: processing times reflect specimen handling, whereas collection time anchors the sample to the patient’s temporal context. Preserving this distinction may be particularly useful for transcriptional, proteomic, metabolic, and immune analyses [4-7].

Experimental transcriptome atlases provide evidence that sampling time can influence measured gene expression. Zhang et al. examined 12 mouse organs and found that 43% of protein-coding genes exhibited circadian expression in at least one organ, with substantial tissue specificity [15]. Mure et al. subsequently profiled 64 tissues in baboons at two-hour intervals over 24 hours, demonstrating widespread tissue-dependent diurnal expression patterns [4]. These findings provide a rationale for retaining collection time in studies of tissue molecular profiles. However, they do not establish the magnitude of temporal variation in human tumors or demonstrate that sampling time changes clinical biomarker classification. Such applications require direct evaluation in the relevant tumor type and assay.

Human tissue data provide complementary evidence. Ruben et al. applied the cyclic ordering by periodic structure (CYCLOPS) algorithm to reconstruct temporal ordering in gene expression datasets covering 13 tissues from 632 human donors, identifying tissue-specific rhythmic expression patterns [5]. This approach illustrates how temporal structure may be investigated when collection-time information is unavailable. However, computational reconstruction from samples obtained from different individuals is not equivalent to prospective repeated sampling within the same person. These findings support the biological rationale for temporal annotation but do not establish the effect of biopsy timing on a particular tumor assay or clinical decision.

Circadian pathways interact with several cancer-related processes. Experimental evidence in humans suggests that circadian misalignment can alter rhythmic expression of genes involved in cancer-related pathways and DNA repair [16]. However, the magnitude and clinical relevance of daily variation remain insufficiently characterized for many routinely assessed tumor biomarkers. Consistent timestamping would support investigation of these effects without assuming that every marker requires time-standardized sampling.

Temporal variation is more readily recognized in blood-based measurements. Circulating leukocyte populations undergo daily redistribution [8-10], which may influence inflammatory indices such as the neutrophil-to-lymphocyte ratio and systemic immune-inflammation index. Sleep, stress, corticosteroids, infection, and recent treatment may also affect these measurements. Comparable sampling schedules or reliable documentation of collection time and clinical context may therefore support longitudinal interpretation.

Liquid biopsy provides a cancer-related example. Diamantopoulou et al. investigated circulating tumor cells in patients with breast cancer and complementary mouse models, reporting greater circulating tumor cell release during the resting phase [11]. The experimental models also demonstrated differences in the metastatic capacity and transcriptional characteristics of cells obtained during rest and activity. The study therefore links sampling time to circulating tumor cell abundance and biological properties in the settings investigated. However, circulating tumor cells and circulating tumor DNA are distinct analytes, and these results do not establish equivalent temporal patterns in circulating tumor DNA or across other cancers. Studies evaluating liquid biopsy timing should assess each analyte separately and determine whether observed variation affects detection, quantification, or interpretation of serial measurements.

Serial samples collected at different hours may also differ with respect to food intake, activity, medication exposure, or sleep-wake timing. Observed changes could consequently reflect several overlapping influences alongside disease evolution or treatment response. Comparable collection windows may reduce this variability when feasible. Otherwise, consistently recorded timestamps provide important context and, where the study design and data permit, support adjustment, stratification, or sensitivity analyses. The proposed minimum temporal metadata for chrono-biobanking and the purpose of each variable are summarized in Table 1.

**Table 1:** Proposed Temporal Metadata for Chrono-Biobanking Proposed temporal metadata for chrono-biobanking. Collection and processing events are recorded separately and linked to specimen identity. Treatment and immediate-context information provide additional context when relevant and available. These data may support time-aware comparisons and evaluation of pre-analytical and biological sources of variability. Collection clock time does not, by itself, establish internal circadian phase.

Temporal domain	Recommended metadata	Purpose	
Specimen collection	Local collection date and time, with the event clearly defined	Provides a time-of-day reference for the collection event	
Time-zone context	Time zone and applicable UTC offset, when available	Supports comparisons across centers and calculation of intervals	
Specimen identity	Specimen identifier, type, and anatomical site	Links the timestamp to the correct specimen and its biological context	
Processing	Times of laboratory receipt and relevant processing or fixation events, when available	Supports assessment of pre-analytical handling intervals	
Timestamp reliability	Source, recording precision, and whether the time is documented, estimated, or unknown	Documents uncertainty and supports data-quality assessment	
Treatment context - enhanced	Date and time of the most recent relevant treatment, when applicable	Allows sampling time to be considered relative to treatment exposure	
Immediate context - enhanced	Fasting status, recent meals, and relevant medication exposure, when available	Provides context for interpreting potentially time-sensitive biomarkers	

Clinical trials and translational studies offer practical settings for implementing temporal annotation. Protocols may specify sampling relative to treatment administration, while the clock time of collection may receive less attention. Recording both would preserve distinct information about treatment exposure and time of day. As noted above, clock time alone does not establish internal circadian phase. Timestamps could initially be collected as exploratory metadata without changing clinical schedules. Biomarker-specific collection windows could subsequently be considered if supported by appropriate evidence. The proposed temporal metadata are summarized in Table 1.

Beyond clinical trials, large observational datasets provide a complementary rationale for recording sampling time in translational studies. Wyse et al. examined seasonal and daytime variation in immune parameters in 329,261 UK Biobank participants [17]. They reported daytime variation in white blood cell counts, including neutrophil, lymphocyte, and monocyte counts, as well as C-reactive protein. Most associations remained statistically significant after adjustment for selected demographic, lifestyle, and environmental factors, although the morning segment of the C-reactive protein curve did not. These findings suggest that collection time may contribute to differences in blood-based immune profiles outside dedicated chronobiological experiments. However, comparisons between individuals sampled at different hours cannot establish within-person circadian rhythms or exclude residual differences in participant characteristics and appointment schedules.

Together, these considerations support the systematic recording of collection time in oncology studies incorporating blood-based immune endpoints. Where the study design and data permit, sampling time can be evaluated alongside treatment exposure, clinical status, and other relevant covariates. This approach may help determine whether unaccounted-for temporal variation could confound the interpretation of biomarker differences attributed to tumor heterogeneity or treatment-associated changes. Such possibilities require direct investigation in the relevant clinical setting and do not, by themselves, justify rigid biopsy schedules.

Relevance to geroscience and longevity medicine

Longevity medicine seeks to preserve healthspan, functional independence, and physiological resilience. Its biomarker approaches include DNA methylation clocks and transcriptomic, proteomic, metabolomic, inflammatory, and physiological measurements [12-14]. Longitudinal multi-omics profiling has also described individual patterns of change across metabolic, immune, hepatic, renal, and other pathways [18].

Although aging-related measurements often assess differences between individuals or changes over months and years, some underlying signals may fluctuate within a day. Circadian regulation influences circulating metabolites, plasma proteins, hormones, immune cell composition, and gene expression [5,19,20]. Sampling time may therefore contribute to variation in certain measurements without necessarily indicating a change in the underlying aging trajectory. The magnitude and relevance of such effects are likely to differ among estimators and require validation for each biological age measure.

Human metabolomic and proteomic studies offer useful examples. In a 40-hour constant-routine protocol with controlled light, posture, hourly isocaloric meals, and extended wakefulness, Dallmann et al. reported that approximately 15% of identified plasma and saliva metabolites showed circadian variation [19]. Depner et al. studied six healthy men under aligned and simulated night-shift conditions and described 24-hour patterns in 573 of 1,129 plasma proteins; circadian misalignment was associated with altered average abundance and/or temporal patterns of 127 proteins [20]. These findings suggest that molecular measurements may reflect endogenous timing, behavioral schedules, and their interaction. However, neither study evaluated biological age estimators, and the small study populations limit extrapolation to other groups, including older adults and patients with cancer.

Recent work in proteomic aging extends this question beyond individual circulating proteins. Oh et al. analyzed plasma proteomic data from 5,676 participants across five cohorts and developed models estimating aging in 11 major organs [21]. Organ-specific age estimates were associated with health and disease outcomes, illustrating the potential of blood-based measurements to capture heterogeneous aging profiles. These results do not establish whether the resulting estimates are sensitive to collection time. Considered alongside experimental evidence of daily variation in the plasma proteome [20], they provide a rationale for directly testing the temporal stability of complete proteomic aging models. Such testing would ideally distinguish changes in individual protein concentrations from changes large enough to influence model output or its clinical interpretation.

For biological age estimators, temporal sensitivity may be most informative when assessed at the level of the complete algorithm rather than inferred solely from variation in individual components. One possible approach would use repeated samples from the same participants across the day, with standardized processing and documentation of sleep, meals, and medications. Analyses could then describe within-day variation in the resulting age estimate and explore whether differences associated with sampling time are large enough to affect longitudinal comparisons or estimated intervention responses. Such work would help distinguish detectable molecular rhythms from variation sufficient to influence interpretation of an aging biomarker.

Sampling time consistency may also be relevant when evaluating longevity interventions. Nutrition, time-restricted eating, exercise, sleep modification, and medications can influence biomarker levels and temporal patterns. Different baseline and follow-up collection times may complicate interpretation of intervention effects, and single-time-point measurements may not capture changes in rhythmic amplitude or phase. Repeated sampling, together with consistent documentation of collection times, would provide the temporal information needed to investigate such changes.

Intervention studies also illustrate why aging biomarkers should be interpreted individually. In a post hoc analysis of the randomized CALERIE trial, Waziry et al. reported that two years of caloric restriction slowed the pace of aging measured by DunedinPACE, whereas PhenoAge and GrimAge did not show statistically significant intervention effects [22]. This divergence suggests that different DNA methylation algorithms need not respond equivalently to the same intervention. It does not indicate that sampling time explained the differences or that the reported effects were circadian in origin. For future trials, a complementary approach would be to assess the repeatability of each selected estimator under comparable collection conditions and, where feasible, to relate within-day variability to the expected intervention effect.

Aging is associated with changes in circadian amplitude, phase, sleep-wake organization, synchronization, and responsiveness to environmental cues [14]. Temporal organization may therefore offer information beyond its potential role as a source of variability. We use the term temporal resilience here to describe the proposed capacity to preserve or restore appropriately timed biological functions during stress, illness, treatment, or aging. Its measurement and clinical relevance remain to be established.

Chrono-biobanking may support longevity research by improving comparability between measurements and enabling investigation of rhythmic changes as potential markers of aging or intervention response. A biomarker's average level and its temporal pattern may provide different information, although the relevance of this distinction requires validation.

Aging biomarker frameworks emphasize validity, reproducibility, sensitivity to change, and clinically meaningful outcomes [23,24]. Evaluating sensitivity to collection time could complement these criteria. A practical first step is systematic timestamping and, where feasible, comparable serial sampling windows. More intensive circadian profiling could be reserved for selected biomarkers or interventions.

Even when timestamps exist in electronic records, they may not be consistently retained in research datasets or distinguished from processing times. The proposed framework addresses this practical gap by preserving clearly defined temporal information.

A practical framework for biopsy timestamping and chrono-biobanking

Chrono-biobanking aims to preserve the temporal context of specimen collection through systematic timestamping and, where feasible and justified, comparable sampling schedules. It is intended to complement existing frameworks addressing collection, ischemia, fixation, processing, and storage [1,2].

It is useful to distinguish the time of specimen collection from the times of laboratory receipt, processing, and storage. Tissue removal or blood sampling provides a reference for the patient's temporal context, whereas subsequent timestamps describe specimen-handling conditions. Both types of information may be relevant to the interpretation of analytical results. These event definitions and the associated minimum metadata are summarized in Table 1.

Existing informatics standards provide a starting point for implementation. The Health Level Seven International Fast Healthcare Interoperability Resources (HL7 FHIR) Specimen resource distinguishes specimen collection time from receipt time and supports documentation of processing events [25]. The proposed framework could build on these distinctions by mapping each recorded timestamp to a clearly defined event across clinical, laboratory, and biobank systems. The availability and completeness of these fields in practice would need to be assessed locally, as implementation varies across institutions and systems.

In multicenter studies, a shared data dictionary would ideally specify event definitions, recording precision, and the handling of time zones and daylight-saving changes. Preserving the original local date and time together with the applicable UTC (Coordinated Universal Time) offset and converting to UTC where possible would support analysis of local time-of-day effects and calculation of intervals between events. Time-zone offsets are particularly important around daylight-saving transitions, when a given local time may occur twice or be skipped. When a timestamp or its time-zone information is unavailable or uncertain, this limitation should be documented rather than a value inferred without supporting information.

Preserving this information would allow sampling-time effects to be examined without altering clinical workflows. Used in this way, chrono-biobanking represents a practical and low-burden step toward improving the comparability and interpretability of biospecimen-based measurements.

Minimum temporal metadata

The proposed minimum dataset, summarized in Table 1, includes the local collection date and time in a standardized 24-hour format, the applicable time zone or UTC offset when available, specimen type, anatomical site, and key processing timestamps. For tissue specimens, collection time would ideally refer to tissue removal whenever reliably documented; for blood specimens, it would reflect the sampling event rather than registration or laboratory receipt. Unknown or estimated times should be identified as such.

Each timestamp would benefit from being accompanied by its source and recording precision. A directly documented collection time should be distinguishable from a retrospectively reconstructed estimate or a time recorded only to the nearest hour. Missing collection times are best explicitly coded as missing rather than replaced with laboratory receipt times. For surgical specimens, the recorded event should be clearly defined because the start of surgery, tissue removal, and placement in fixative are separate events. When multiple specimens are obtained during the same procedure, specimen-specific collection times should preferably be retained whenever feasible.

A tiered approach could accommodate different clinical and research settings, reflecting increasing resource requirements and decreasing generalizability:

Essential annotation includes specimen identity, collection date and time, the applicable time zone or UTC offset when available, and key processing timestamps. Electronic and barcode systems may facilitate capture, provided that recorded events are clearly defined and correspond to actual sampling or handling.

Enhanced annotation may include time since awakening, fasting status, recent meals, relevant medications, and treatment timing, particularly in prospective or longitudinal studies.

Comprehensive annotation may incorporate chronotype, sleep schedules, shift work, recent travel, light exposure, actigraphy, and validated circadian-phase measures in dedicated chronobiological investigations.

For serial sampling, comparable collection windows and treatment-relative intervals may improve interpretation when feasible. Actual collection times and relevant deviations from planned schedules should also be retained. Electronic systems could store these data as structured fields linked to specimen identifiers, with timestamp sources and reliability documented.

Temporal annotation should remain linked to specimen identity as material moves through the biobank. The Minimum Information About BIobank Data Sharing (MIABIS) terminology includes individual-level components describing samples, sample donors, and events, providing a structured terminology for describing biospecimens and their collection context [26]. Within the proposed framework, aliquots and derived materials should retain a traceable relationship to the original collection event, while subsequent processing events are recorded separately. For example, RNA extracted several days after a biopsy should remain associated with the biopsy collection time, as well as the extraction date and relevant handling intervals. This linkage would help preserve temporal information when specimens are subdivided, transferred between institutions, or converted into molecular datasets.

Implementation could begin with an audit of existing records to determine whether collection and processing times are separately available and consistently linked to specimen identifiers. Prospective workflows could then introduce structured fields for the collection event, timestamp source, and relevant deviations from the planned sampling schedule. Quality checks could help flag missing values and implausible event sequences, such as laboratory receipt preceding documented collection. Such checks would assess documentation quality without assuming that a recorded timestamp accurately represents the sampling event.

A pilot implementation could evaluate operational feasibility separately from biological utility. Initial outcomes could include the proportion of specimens with a documented collection time, agreement between electronic timestamps and source records, successful linkage to treatment events, and the additional staff time required for annotation. A subsequent biomarker-focused phase could test whether temporal metadata help explain variability beyond established clinical and preanalytical factors or improve the precision of longitudinal comparisons. These outcomes would distinguish better documentation from demonstrated improvements in biomarker interpretation. Expansion to more detailed annotation or standardized collection windows would depend on the research question, the observed benefit, and the burden on patients and clinical services.

Challenges and future directions

Clock time provides a practical temporal reference but does not directly measure internal circadian phase. Chronotype, sleep, shift work, travel, illness, medications, and hospitalization may influence the relationship between the two. Additional measures of circadian phase may therefore be useful when required by the research question.

Molecular approaches may help bridge the gap between collection clock time and internal circadian phase. Wittenbrink et al. developed a blood transcript-based assay for estimating internal circadian time from a single sample, demonstrating the feasibility of molecular phase assessment under the conditions studied [27]. Such approaches could complement timestamping in selected investigations, but their performance would need to be established in the intended population before routine application. In oncology, validation would need to consider whether malignancy, treatment exposure, altered blood cell composition, or disrupted sleep affects phase estimation. Moreover, a blood-derived phase estimate should not be assumed to represent the phase of every peripheral tissue or tumor.

Temporal effects differ across biomarkers, tissues, individuals, and analytical platforms. Aging-related changes in circadian organization [14] and disease-related disruption may limit extrapolation from healthy populations. Biomarker-specific validation is therefore needed before fixed collection windows can be recommended.

Retrospective timestamps may be missing, rounded, entered later, or confused with laboratory receipt times. Studies would benefit from describing their source, completeness, and reliability, and from acknowledging uncertainty when collection windows are reconstructed.

Missing temporal information may also introduce selection bias if documentation quality differs systematically between clinical settings. For example, complete collection times may be more readily available for scheduled outpatient procedures than for emergency or externally obtained specimens. Analyses restricted to fully timestamped samples could therefore select a clinically different population. Studies would benefit from reporting timestamp completeness by center, specimen type, and relevant patient characteristics, and from comparing included with excluded samples where possible. Sensitivity analyses using alternative assumptions about uncertain collection windows, for example, treating rounded times as interval-censored, may help assess robustness, but reconstructed or imputed times should remain distinguishable from directly observed timestamps.

Clinical feasibility also warrants attention. Documentation should fit existing workflows without delaying care or limiting access. Standardized collection windows may be considered when evidence supports a meaningful improvement in biomarker interpretation or comparability.

Future research could begin by examining associations between collection time and biomarker variability in existing datasets, followed by prospective repeated-sampling studies. Analyses would ideally account for the possibility that sampling time is associated with patient characteristics or clinical workflows. Morning and afternoon samples may differ in fasting status, treatment exposure, inpatient or outpatient setting, or procedural indication. An association between clock time and a biomarker therefore does not, by itself, establish endogenous circadian regulation.

Repeated-sampling designs can help distinguish within-person temporal variation from between-person differences, provided that analyses account for correlated measurements and other relevant sources of variability. Where the sampling schedule provides adequate coverage, time of day may be modeled as a continuous cyclic variable rather than divided into arbitrary morning and afternoon categories. Findings would ideally include effect estimates and measures of uncertainty and assess whether observed variation could affect a clinically relevant classification or interpretation.

Study design would ideally match the temporal feature being investigated. A single specimen per participant may support analysis of population-level time-of-day associations, but it cannot characterize that individual's rhythmic amplitude, phase stability, or recovery after disruption. Guidelines for genome-scale rhythm analysis emphasize the importance of sampling design, replication, and appropriate statistical evaluation [28]. Prospective chrono-biobanking studies would therefore benefit from providing sufficient temporal coverage for the intended analysis. Laboratory processing order and analytical batches should also be balanced across collection times where feasible; otherwise, technical variation may be mistaken for a biological temporal pattern. Exploratory findings would ideally undergo independent validation, with appropriate control of multiple testing when many molecular features are assessed.

In geroscience, sampling-time sensitivity could complement established criteria for reproducibility, responsiveness, and clinical relevance [23,24]. Studies could also explore whether rhythmic amplitude, phase, or recovery after circadian disruption adds information beyond single-time-point biological age estimates.

Clearly defined collection timestamps could complement existing preanalytical metadata. Electronic systems may facilitate consistent documentation, while wearable data could support additional temporal characterization in selected studies. Together, these approaches would provide a basis for evaluating whether, for which biomarkers, and under what conditions sampling time is clinically relevant.

Limitations

This article is a narrative review, and its interpretation is subject to the limitations of the literature selection and synthesis approach. The evidence was not assessed through a formal systematic review or quantitative meta-analysis; therefore, the review does not provide pooled estimates or a comprehensive assessment of the strength of evidence across studies. The selection of publications may also have been influenced by the scope of the review and the authors’ judgment. The search was limited to English-language publications, and literature published after September 1, 2026, was outside the stated publication period. Relevant non-English literature and more recent evidence may therefore have been missed. No formal assessment of publication bias was performed.

The studies discussed differ in design, populations, specimen types, biomarkers, sampling schedules, and analytical methods, as well as in whether sampling time was reported. These differences limit direct comparisons and the generalizability of findings. Evidence from experimental models and healthy volunteers may not translate directly to patients with cancer, older adults, or routine clinical settings. In addition, time-of-day associations do not necessarily establish endogenous circadian regulation.

The proposed chrono-biobanking framework is a conceptual and practical proposal rather than a validated clinical standard. Its feasibility, resource requirements, burden on clinical and laboratory workflows, and effect on biomarker interpretation require evaluation in real-world settings. Likewise, the temporal sensitivity of individual biomarkers and complete biological-age estimators should not be assumed without direct assessment.

Nevertheless, the review synthesizes evidence across several relevant domains and offers a practical framework that can be tested empirically. Its main contribution is to make the temporal dimension of biospecimen collection explicit and, where feasible, actionable.

## Conclusions

Specimen collection time provides biological context that may be incompletely documented or inconsistently retained in research datasets. More reliable recording could support the investigation of variability in molecular profiles, circulating biomarkers, and longitudinal assessments in precision oncology, geroscience, and longevity medicine. Current evidence does not support a universal collection hour: temporal sensitivity varies across biomarkers and clinical settings and requires biomarker-specific validation. Consistent timestamping offers a practical foundation for evaluating these effects.

Building on this, chrono-biobanking proposes systematic documentation of collection time and, where feasible and scientifically justified, standardization of sampling schedules. Collection date and local time should be clearly distinguished from receipt, processing, fixation, and storage timestamps, with additional information on sleep, fasting, medications, and treatment timing recorded where relevant. Integration into existing workflows could support more comparable and interpretable specimen comparisons without delaying necessary care. Preserving this temporal context could support reproducibility and improve the interpretability of biomarker measurements as evidence develops.
